# Quorum Sensing Activity of *Serratia fonticola* Strain RB-25 Isolated from an Ex-landfill Site

**DOI:** 10.3390/s140305136

**Published:** 2014-03-12

**Authors:** Robson Ee, Yan-Lue Lim, Kok-Keng Tee, Wai-Fong Yin, Kok-Gan Chan

**Affiliations:** 1 Division of Genetics and Molecular Biology, Institute of Biological Sciences, Faculty of Science, University of Malaya, Kuala Lumpur 50603, Malaysia; E-Mails: robsonee@live.com (R.E.); yanluelim@hotmail.com (Y.-L.L.); yinwaifong@yahoo.com (W.-F.Y.); 2 Centre of Excellence for Research in AIDS (CERiA), Department of Medicine, Faculty of Medicine, University of Malaya, Kuala Lumpur 50603, Malaysia; E-Mail: k2tee@um.edu.my

**Keywords:** cell-to-cell communication, matrix-assisted laser desorption ionization time-of-flight (MALDI-TOF), mass spectrometry, triple quadrupole liquid chromatography mass spectrometry, *N*-butyryl-L-homoserine lactone (C4-HSL), *N*-hexanoyl-L-homoserine lactone (C6-HSL), *N*-(3-oxohexanoyl) homoserine-lactone (3-oxo-C6 HSL)

## Abstract

Quorum sensing is a unique bacterial communication system which permits bacteria to synchronize their behaviour in accordance with the population density. The operation of this communication network involves the use of diffusible autoinducer molecules, termed *N*-acylhomoserine lactones (AHLs). *Serratia* spp. are well known for their use of quorum sensing to regulate the expression of various genes. In this study, we aimed to characterized the AHL production of a bacterium designated as strain RB-25 isolated from a former domestic waste landfill site. It was identified as *Serratia fonticola* using matrix-assisted laser desorption ionization time-of-flight (MALDI-TOF) mass spectrometry analysis and this was confirmed by 16S ribosomal DNA sequencing. High resolution triple quadrupole liquid chromatography-mass spectrometry analysis of *S. fonticola* strain RB-25 spent culture supernatant indicated the existence of three AHLs namely: *N*-butyryl-L-homoserine lactone (C4-HSL), *N*-hexanoyl-L-homoserine lactone (C6-HSL) and *N*-(3-oxohexanoyl) homoserine-lactone (3-oxo-C6 HSL). This is the first report of the production of these AHLs in *S. fonticola.*

## 1. Introduction

*Serratia* spp. are well known for the production of prodigiosin [1]. As part of the Enterobacteriaceae family, *Serratia* spp. are frequently reported to be the cause of food spoilage [1]. The ubiquitous nature of bacteria from this genus enables their survival in diverse habitats, including host digestive tracts, soil and water [2]. For example, while the majority of *Serratia* spp. are well-known for their role as a plant-growth promoting rhizobacterium, a large number of *Serratia* spp. are also repeatedly found to be isolated from respiratory tract and urinary tract clinical samples [3–6]. Due to its growing clinical significance as well as its beneficial environmental contributions, a more comprehensive investigation on the physiological properties and in particular the quorum-sensing behaviour of bacteria of this genus is crucial [7,8].

Quorum sensing (QS) is an interaction mechanism employed by unicellular bacteria to achieve cell-to-cell communication in order to regulate expression of various cell density-dependent genes [9–11]. QS is achieved by bacteria *via* production of and response upon detection to various QS signaling molecules termed autoinducers. These signaling mechanisms enable the bacterial population to maintain the cell density within individual species as well as among other species in the surrounding environment via detection of accumulated concentrations of exogenous signaling molecules, and subsequently allow regulation of the expression of diverse beneficial genes. Examples of QS regulated activities reported are biofilm formation and swarming activities in *Pseudomonas aeruginosa* [12,13], antibiotic production in *Erwinia carotovora* [14], and Ti plasmid conjugation in *Agrobacterium tumefaciens* [15]. To date, the most extensively explored QS molecules are the *N*-acylhomoserine lactones (AHLs), diffusible signaling molecules whose structure comprises a five-membered lactone ring that contains an amide side chain of varying length. [16]. To understand the virulence, antibiotic susceptibility, physiology and metabolism of *S. fonticola* one should start primarily by characterizing its AHL expression [17].

In this study, we isolated a strain RB-25 identified as *S. fonticola* from a site formerly used as domestic waste dumping site. Identification of the isolate was done by using matrix-assisted laser desorption ionization time-of-flight (MALDI-TOF) mass spectrometry analysis and sequence analysis of 16S ribosomal DNA (rDNA). This isolate triggered both *Chromobacterium violaeum* CV026 and *E. coli* [pSB401] biosensors. Subsequently, its AHL profile was confirmed unequivocally by using high resolution quadrupole mass spectrometry. The former landfill site was selected as the sampling site as there is limited documentation about the microbial population with QS properties in this unique environment.

## 2. Experimental Section

### 2.1. Strain Isolation, Enrichment and Condition

A former dumping ground in Ayer Hitam (Malaysia; GPS coordinates N03′00′12.1 E101′39′33′1) was selected as the sampling site. Soil specimens were collected at a subsurface depth of 10 cm using a clean 50 mL Falcon tube. Subsequently, soil samples were inoculated into KGm medium and enrichment was done for four cycles [18,19]. In short, soil samples were mixed thoroughly with 10 mL of KGm medium. Subsequent inoculation of soil suspension into fresh KGm medium was performed every 48 h and the inoculum was incubated with agitation. The KGm medium used was supplied with 50 mM 3-oxo-C6-HSL (Sigma-Aldrich, St. Louis, MO, USA). A diluted suspension was then spread on a plate of KGm agar supplemented with 3-oxo-C6-HSL and culture was spread on Luria-Bertani (LB) agar in the last enrichment cycle. The sole growth media used throughout the entire experiment are LB broth and LB agar. All strains were cultured aerobically in 28 °C with exception of *E. coli* [pSB401], which was cultured in 37 °C.

### 2.2. AHL Production Screening

Short chain AHLs production was screened by using two different biosensors namely *C. violaceum* CV026 and *E. coli* [pSB401]. The *C. violaceum* CV026 biosensor responds to short chain AHLs by producing a purple pigmentation after an overnight incubation at 28 °C. AHL activity was then re-confirmed with a bioluminescence assay involving the use of the *lux*-based biosensor *E. coli* [pSB401]. Positive and negative controls for short chain AHLs detection were *E. carotovora* GS101 and *E. carotovora* PNP22, respectively. All reporter strains and both positive and negative control strains (Table 1) were gifts kindly provided by Professor Paul Williams (University of Nottingham, Nottingham, UK) (Table 1).

### 2.3. Extraction of AHLs

Isolates that showed QS activity were cultured in LB medium buffered to pH 5.5 with 50 mM (MOPS) in order to avoid alkaline-pH-driven deterioration of any AHLs. Log phase culture was subsequently extracted twice *via* the solvent extraction method. The solvent utilised in this extraction procedure was ethyl acetate supplemented with 0.1% glacial acetic acid [20]. The dried extracts were kept at −20 °C for storage purposes.

### 2.4. Bioluminescence Analysis

Measurement of bioluminescence were determined by using an Infinite M200 luminometer (Tecan, Männerdorf, Switzerland). Dessicated AHL extracts were resuspended with *lux*-based *E. coli* [pSB401] biosensor (OD_600_ of 0.1) before dispensing into each well of 96-microtitre well plates in triplicate and the experiments were independently repeated three times. Bioluminescence activities were analyzed as luminescence (relative light unit) at the excitation wavelength of 495 nm (RLU/OD_495nm_) over the course of 24 h. Reading of optical density and bioluminescence were automatically and simultaneously recorded at 60 min intervals [18,21].

### 2.5. Assay of AHLs Using Liquid Chromatography Triple Quadrupole Mass Spectrometry (LC-MS/MS)

AHLs produced by the QS isolate were studied using a High Resolution Tandem Liquid Chromatography Quadrupole Mass Spectrometry (LC-MS/MS; Agilent 1290 Infinity LC and Agilent 6490 Triple Quadrupole LC/MS systems, Agilent Technologies Inc., Santa Clara, CA, USA) as described previously [18,19]. The column used was Agilent Zorbax Rapid Resolution High Definition SB-C18 Threaded Column. Precursor ion scan mode was used for AHL detection where the product ion *m*/*z* value was fixed at *m*/*z* 102. Spectra analysis using the Agilent Mass Hunter software was performed as reported [18,19].

### 2.6. Matrix-Assisted Laser Desorption Ionization Time-of-Flight Mass Spectrometry (MALDI-TOF MS) for Strain Identification

MALDI TOF MS was performed essentially as reported previously [18,19]. Bacterial samples were smeared very thinly on the MSP 96 target polished steel BC plate and MALDI matrix solution was added. A Microflex MALDI-TOF (Bruker Daltonik GmbH, Leipzig, Germany) bench-top mass spectrometer was used for identification of sample isolates [18,19,22] and for analysis of the MALDI-TOF mass spectra, we used the version 3.1 (Build 65) of the Bruker MALDI Biotyper Real Time Classification Classification software. To design the dendrogram for bacteria identification, we then used the MALDI Biotyper MSP creation method (Bruker Daltonics, Bremen, Germany) as reported previously [18,19,22].

### 2.7. Molecular Identification by 16S Ribosomal DNA (16S rDNA) Sequencing

To amplify the 16S rDNA genes, we used the reported method [23] with the following universal primers: 27F forward primers, 515F forward primers and 1525R reverse primers. Purification of genomic DNA, 16S rDNA PCR template and PCR condition was performed as reported elsewehere [23] and a phylognetic tree was conducted using MEGA5 [24].

## 3. Results and Discussion

### 3.1. Ioslation of Soil Bacteriaand Screening of Their AHLs Production

**Figure 1.**
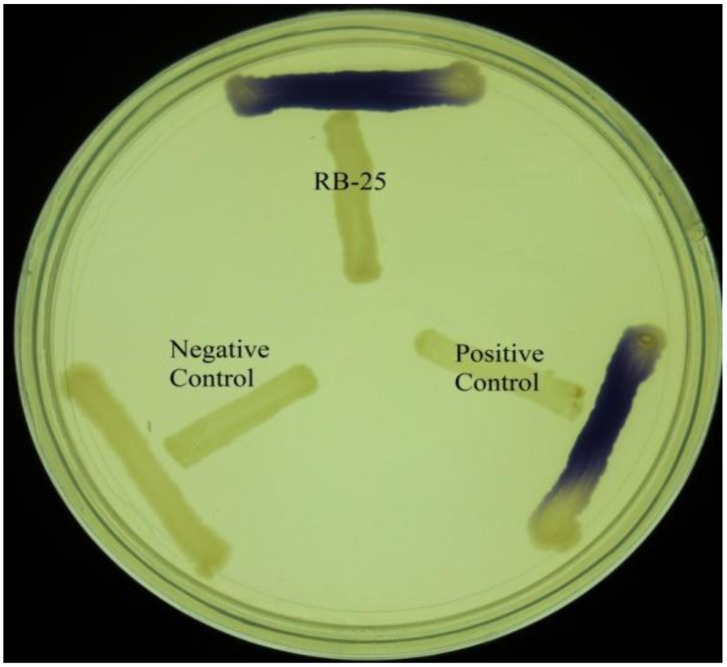
AHL production by strain RB-25. Purple pigmention observed in *C. violaceum* CV026 after overnight incubation when strain RB-25 was cross streaked with CV026 colony. *E. carotovora* GS101 was included as positive control while *E. carotovora* PNP22 was included as negative controls.

KGm medium is a minimal medium which facilitates growth of non-fastidious bacteria and quorum quenching bacteria [18,19]. Soil samples were inoculated into sterile KGm medium and four cycles of enrichment was conducted. It was noted that KGm medium became turbid after two days. This indicated growth of microorganisms during each enrichment cycle. No growth was detected under our present experimental condition in the control KGm medium where AHL was not supplemented (data not shown). Bacterial isolates were streaked in several passages in order to obtain single, pure colonies and the bacteria were examined for both AHL production and degradation using the two types of biosensors. For screening of AHL activities, a number of AHL biosensors have been constructed, for instance *C. violaceum* CV026 was used for screening of short chain AHL production [21,25,26]. This AHL biosensor relies on the Cvi-based QS system whereby violacein is produced as a result of the presence of short chain AHLs. This makes CV026 a very convenient biosensor for initial and rapid screening of AHLs based on pigment formation [25]. Based on this preliminary screening, we detected that strain RB-25 produced short chain AHLs (Figure 1). However no significant degradation of AHLs was observed, suggesting strain RB-25 is a non-fastidous bacteria capable of thriving under nutrient limiting conditions.

**Figure 2.**
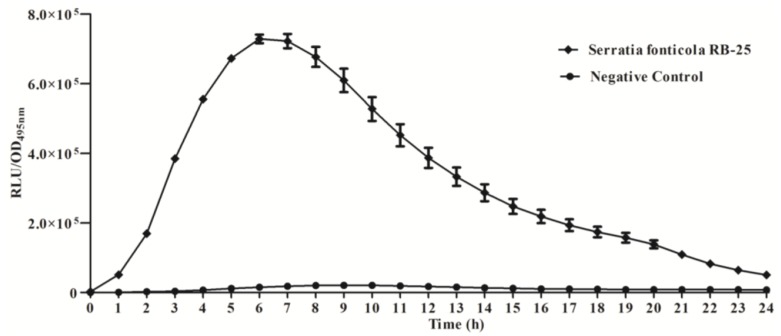
Detection of short chain AHLs produced by isolate RB-25 *via E. coli* [pSB401] biosensor. Three independent experiments were conducted and each point is the mean of these results. *E. coli* [pSB401] (diamond), negative control (circle) (extract obtained from un-inoculated LB broth).

We further studied the production of AHLs using bioluminescence analysis with *E. coli* [pSB401] biosensor cells which detect short chain AHLs [21, 26], confirming that strain RB-25 produced short chain AHLs (Figure 2). Production of short chain AHLs was seen as the increment of RLU/OD_495nm_ value observed over 24 h.

### 3.2. Molecular and MALDI-TOF Identification of Strain RB-25

**Figure 3.**
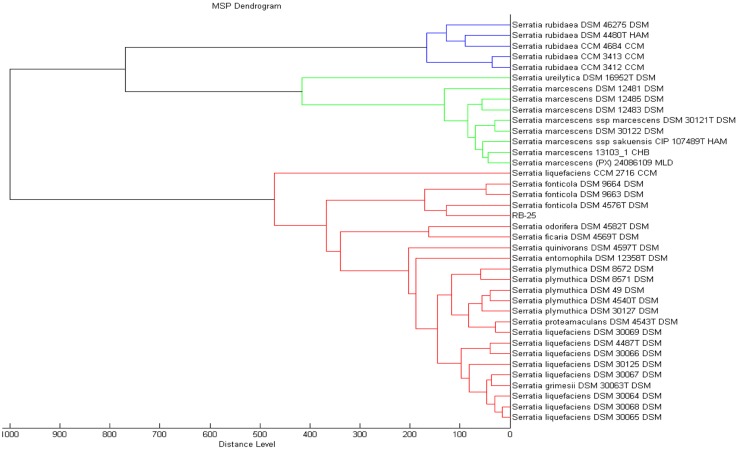
Dendrogram demonstrating identification of strain RB-25. MALDI-TOF mass spectra analysis indicating strain RB-25 clustered within the species of *S. fonticola*.

MALDI-TOF has been proven to be a rapid and reliable technique useful for bacteria biotyping [27–29]. In the present work, preliminary identification was done using MALDI-TOF where we could identified isolate RB-25 as *S. fonticola* with a value of 2.361, which indicated a reliable detection at both the genus and species level. To obtain confident identification at the species level, the MALDI-TOF scoring system value should be higher than 2.3 (Figure 3) [18,19,22].

**Figure 4.**
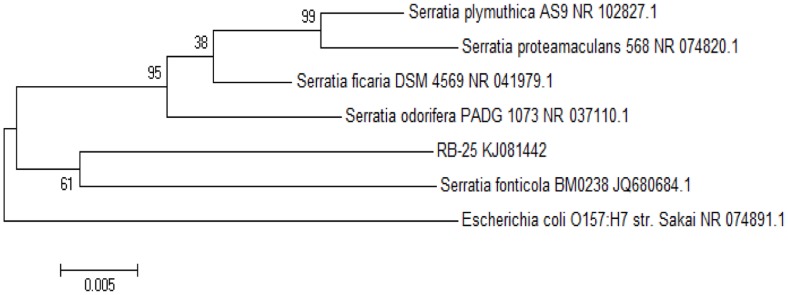
Phylogenetics analysis of strain RB-25.A total of 1,357 positions in the final dataset was used where only bootstrap values >50% are shown at branches. Bar: 5 substitutions per 1,000 nucleotides. GenBank accession number for *S. fonticola* strain RB-25 is KJ081442.

To further confirm the identity of strain RB-25, we then used a molecular approach by analyzing its 16S rDNA gene nucleotide sequences using phylogenetics. Web-based comparison against the NCBI database and phylogenetic analysis of 16S rDNA sequences of strain RB-25 confirmed that this isolate is indeed *S. fonticola* (Figure 4). In this work, the Neighbor-Joining method [30] with bootstrap value from 1,000 replicates [31] and Maximum Composite Likelihood method [32] were used.

### 3.3. Identification of AHLs

AHLs production has been documented in some species of *Serratia* where the most frequently detected AHL is C6-HSL [1]. Production of C4-HSL and C6-HSL had also been documented in *Serratia* sp. ATCC 39006 [33] and *S. marcescens* MG1 [34,35], whereas some *Serratia* spp. like *S. plymuthica* RVH1, *S. marcescens* SS-1 and *S. proteamaculans* B5a have been reported to produce other types of AHLs [8,36,37].

**Figure 5.**
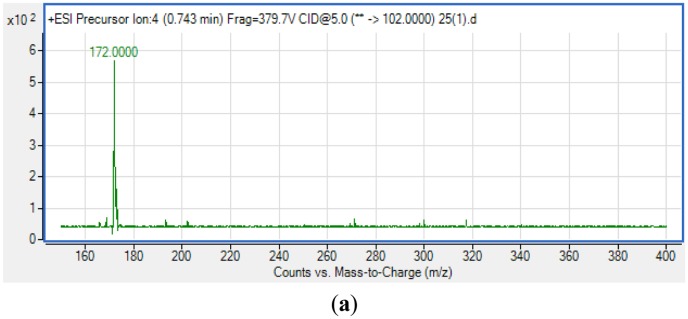

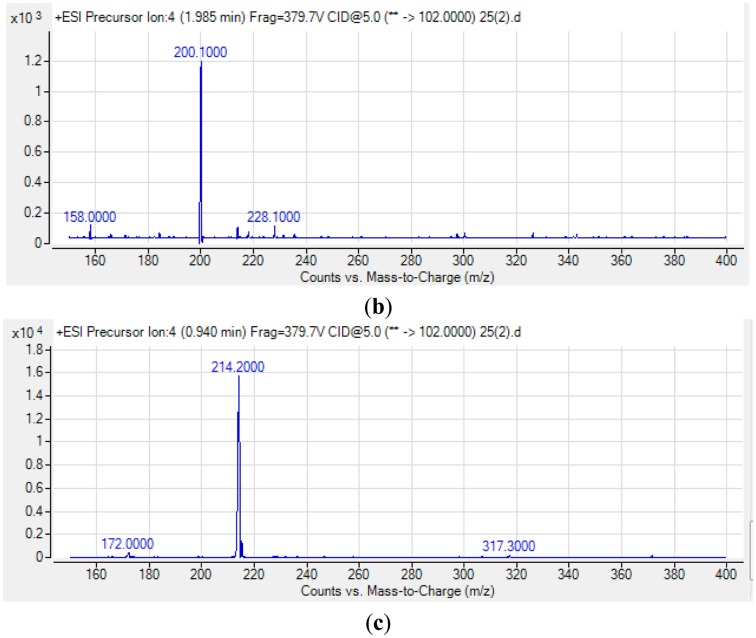
Mass spectrometry analysis of AHLs produced by *Serratia fonticola* RB-25. Mass spectra show that *S. fonticola* produces C4-HSL (**a**) (retention time: 0.743 min; Abundance: 556.7; Abundance %: 100; *m*/*z*: 172.0000); C6-HSL (**b**) (retention time: 1.985 min; Abundance: 1198.12; Abundance %: 100; *m*/*z*: 200.1000) and 3-oxo-C6-HSL (**c**) (retention time: 0.940 min; Abundance: 15,467.14; Abundance %: 100; *m*/*z*: 214.2000).

For *S. fonticola*, although its QS genes had been documented for rapid identification purposes [38], little is known about its AHL profile. Hence, in this work, we strived to identify if this isolate produced any detectable AHLs. Characterization of its AHLs profile is the first step in understanding the QS properties of *S. fonticola* and its AHL extract was assayed *via* tandem mass spectrometry platform that confirmed the presence of three AHLs, namely C4-HSL, C6-HSL and 3-oxo-C6 HSL (Figure 5).

## 4. Conclusions

In this work, we studied the AHL profile of *S. fonticola* strain RB-25 isolated from a former landfill site. In this paper we confirmed for the first time the presence in the spent supernatant of this strain of three short chain AHLs, namely C4-HSL, C6-HSL and 3-oxo-C6 HSL, thus providing evidence of quorum sensing activity in this isolate.

## Figures and Tables

**Table 1.** Reporter strains and control strains used in this study.

**Strain**	**Description**
*C. violaceum* CV026	Biosensor that produces purple coloured violacein in response to short chain AHLs.
*E. coli* [pSB401]	Biosensor that produces bioluminescence in exposure to short chain AHLs.
*E. carotovora* GS101	Positive control for biosensors. Produces short chain AHLs to activate *C. violaceum* CV026 and *E. coli* [pSB401].
*E. carotovora* PNP22	Negative control for biosensors.
